# Supplementary material for: The use of virtual nominal groups in healthcare research: An extended scoping review
Source: PLoS One. 2024 Jun 12;19(6):e0302437. doi: 10.1371/journal.pone.0302437 (PMC11168680; doi:10.1371/journal.pone.0302437)
Supplement: S2 File — (DOCX) [file pone.0302437.s002.docx]

Appendix 2

S2 File - Author Survey

1. Which virtual platform did you use ?

[ ] Google Hangouts

[ ] Microsoft Teams

[ ] Skype

[ ] Zoom

[ ] Other (please specify): _______

2. For each step of the Nominal Group Technique (NGT) please list if you used a virtual platform or Face to face

Idea generation

[ ] Virtual

[ ] Face-to Face

[ ] other (please specify): ______

Sharing of ideas (usually round robin)

[ ] Virtual

[ ] Face-to Face

[ ] other (please specify): ______

Discussion/clarification

[ ] Virtual

[ ] Face-to Face

[ ] other (please specify): ______

Voting

[ ] Virtual (specify if different than above) ________

[ ] Face-to Face

[ ] other (please specify): ______

3. Which of the following functions did you use?

[ ] Annotations

[ ] Chat

[ ] Polls

[ ] Other (please specify): _______

4. What modifications did you make to the NGT, if any, to accommodate the virtual format?

[ ] none

[ ] other (please specify) __________________

5. Why did you use a virtual format for your Nominal Group Technique ? (select as many as apply)

[ ] COVID restrictions for face-to-face meetings

[ ] COVID travel restrictions

[ ] Decision had nothing to do with COVID

[ ] Allows for participants from different geographic regions

[ ] Other (please specify) ____________________

6. Overall, what are your general impressions of how the virtual platform worked ?

[ ] overall performed similarly to face-to-face

[ ] overall better than face-to-face

[ ] overall not as good as face-to-face

Comments _____________________________________________________________

7. What were the benefits of using a virtual NGT ?

[ ] save money

[ ] save travel time and expense

[ ] allowed for increased choice when selecting of participants

[ ] other _______________________________________________________________

8. What were challenges encountered and how did you manage these ?

[ ] participants not familiar with platform/technology

[ ] technology issues

[ ] reduced participant engagement

[ ] challenges moderating

Comments

_____________________________________________________________

9. If you were to compare your experience with face to face compared to the virtual NGT what would you consider are the most important differences?

________________________________________________________________________

10. If someone were new to the virtual NGT, what suggestions or lessons learned would you share with them?

_______________________________________________________________________

11. Would you be willing to be contacted in the future for a focus group to consider the pros and cons of the virtual NGT ?

[ ] yes

[ ] no

Thank you for completing the survey !
