# Supplementary material for: The use of virtual nominal groups in healthcare research: An extended scoping review
Source: PLoS One. 2024 Jun 12;19(6):e0302437. doi: 10.1371/journal.pone.0302437 (PMC11168680; doi:10.1371/journal.pone.0302437)
Supplement: Attachment — Submitted filename: vNGT Author Response Doc.docx [file pone.0302437.s004.docx]

| Article reference number | PONE-D-23-20161. The Use of Virtual Nominal Groups in Research: An Extended Scoping Review |
| --- | --- |
| Date | December 16^th^, 2023 |

| ***Journal Requirements*** | | |
| --- | --- | --- |
| *Editor comment* | *Authors’ response* | *Location in manuscript (page, line number(s))* |
| 1. Please ensure that your manuscript meets PLOS ONE's style requirements, including those for file naming. The PLOS ONE style templates can be found at  [https://journals.plos.org/plosone/s/file?id=wjVg/PLOSOne_formatting_sample_main_body.pdf](https://can01.safelinks.protection.outlook.com/?url=https%3A%2F%2Fjournals.plos.org%2Fplosone%2Fs%2Ffile%3Fid%3DwjVg%2FPLOSOne_formatting_sample_main_body.pdf&data=05%7C01%7Capace2%40uottawa.mail.onmicrosoft.com%7C00ef3cf4b7554c5848ae08dbe56b5a11%7Cd41fdab17e154cfdb5fa7200e54deb6b%7C1%7C0%7C638356021626035745%7CUnknown%7CTWFpbGZsb3d8eyJWIjoiMC4wLjAwMDAiLCJQIjoiV2luMzIiLCJBTiI6Ik1haWwiLCJXVCI6Mn0%3D%7C3000%7C%7C%7C&sdata=TEaCts1U66hY%2BzxEqyBXIPcfncVYqF%2BgVCRpdq69Gzw%3D&reserved=0) and  [https://journals.plos.org/plosone/s/file?id=ba62/PLOSOne_formatting_sample_title_authors_affiliations.pdf](https://can01.safelinks.protection.outlook.com/?url=https%3A%2F%2Fjournals.plos.org%2Fplosone%2Fs%2Ffile%3Fid%3Dba62%2FPLOSOne_formatting_sample_title_authors_affiliations.pdf&data=05%7C01%7Capace2%40uottawa.mail.onmicrosoft.com%7C00ef3cf4b7554c5848ae08dbe56b5a11%7Cd41fdab17e154cfdb5fa7200e54deb6b%7C1%7C0%7C638356021626035745%7CUnknown%7CTWFpbGZsb3d8eyJWIjoiMC4wLjAwMDAiLCJQIjoiV2luMzIiLCJBTiI6Ik1haWwiLCJXVCI6Mn0%3D%7C3000%7C%7C%7C&sdata=jhkeq9qS14Cguz%2Bn0yrNVoa8lRfslh7PWtqmx7ApaO4%3D&reserved=0) | Yes, this has been completed. | Entire manuscript |
| 2. Thank you for stating the following in the Acknowledgments Section of your manuscript:  "Dr. Humphrey-Murto would like to acknowledge the work of Amanda Pace and Kate Scowcroft from the Department of Innovation in Medical Education for assisting with the study. This project was awarded a Department of Medicine Education Research Grant and a Faculty of Medicine Summer Studentship Award for Seung Ho Lee from the University of Ottawa."  We note that you have provided additional information within the Acknowledgements Section that is not currently declared in your Funding Statement. Please note that funding information should not appear in the Acknowledgments section or other areas of your manuscript. We will only publish funding information present in the Funding Statement section of the online submission form.  Please remove any funding-related text from the manuscript and let us know how you would like to update your Funding Statement. Currently, your Funding Statement reads as follows:  "This project is being funded by a medical education research grant, Department of Medicine, University of Ottawa. The funders will not have any role in the study design, data collection, and analysis, decision to publish or preparation of the manuscript. "  Please include your amended statements within your cover letter; we will change the online submission form on your behalf. | We have removed funding information from the Acknowledgments Section and we have updated our funding statement in the cover letter. | Acknowledgments Section and cover letter |
| 3. Please include your full ethics statement in the ‘Methods’ section of your manuscript file. In your statement, please include the full name of the IRB or ethics committee who approved or waived your study, as well as whether or not you obtained informed written or verbal consent. If consent was waived for your study, please include this information in your statement as well | This was included in the published protocol, but we have also added to this manuscript. | Methods section first paragraph |
| 4. We note that you have indicated that data from this study are available upon request. PLOS only allows data to be available upon request if there are legal or ethical restrictions on sharing data publicly. For more information on unacceptable data access restrictions, please see [http://journals.plos.org/plosone/s/data-availability#loc-unacceptable-data-access-restrictions](https://can01.safelinks.protection.outlook.com/?url=http%3A%2F%2Fjournals.plos.org%2Fplosone%2Fs%2Fdata-availability%23loc-unacceptable-data-access-restrictions&data=05%7C01%7Capace2%40uottawa.mail.onmicrosoft.com%7C00ef3cf4b7554c5848ae08dbe56b5a11%7Cd41fdab17e154cfdb5fa7200e54deb6b%7C1%7C0%7C638356021626035745%7CUnknown%7CTWFpbGZsb3d8eyJWIjoiMC4wLjAwMDAiLCJQIjoiV2luMzIiLCJBTiI6Ik1haWwiLCJXVCI6Mn0%3D%7C3000%7C%7C%7C&sdata=rktd4S7GFN2cfSELdHV%2BCeF7X2aGpNbOiqBUEnSXGJQ%3D&reserved=0).  In your revised cover letter, please address the following prompts:  a) If there are ethical or legal restrictions on sharing a de-identified data set, please explain them in detail (e.g., data contain potentially sensitive information, data are owned by a third-party organization, etc.) and who has imposed them (e.g., an ethics committee). Please also provide contact information for a data access committee, ethics committee, or other institutional body to which data requests may be sent.  b) If there are no restrictions, please upload the minimal anonymized data set necessary to replicate your study findings as either Supporting Information files or to a stable, public repository and provide us with the relevant URLs, DOIs, or accession numbers. For a list of acceptable repositories, please see [http://journals.plos.org/plosone/s/data-availability#loc-recommended-repositories](https://can01.safelinks.protection.outlook.com/?url=http%3A%2F%2Fjournals.plos.org%2Fplosone%2Fs%2Fdata-availability%23loc-recommended-repositories&data=05%7C01%7Capace2%40uottawa.mail.onmicrosoft.com%7C00ef3cf4b7554c5848ae08dbe56b5a11%7Cd41fdab17e154cfdb5fa7200e54deb6b%7C1%7C0%7C638356021626348251%7CUnknown%7CTWFpbGZsb3d8eyJWIjoiMC4wLjAwMDAiLCJQIjoiV2luMzIiLCJBTiI6Ik1haWwiLCJXVCI6Mn0%3D%7C3000%7C%7C%7C&sdata=BqCfw8AHznpOmnQqBB17jKA56PfFuagPAICKxKauI0c%3D&reserved=0).  We will update your Data Availability statement on your behalf to reflect the information you provide | All data from the data extraction is provided in the paper.  Please see revised cover letter that addresses data sharing. |  |
| 5. We note that you have stated that you will provide repository information for your data at acceptance. Should your manuscript be accepted for publication, we will hold it until you provide the relevant accession numbers or DOIs necessary to access your data. If you wish to make changes to your Data Availability statement, please describe these changes in your cover letter and we will update your Data Availability statement to reflect the information you provide. | We have updated the cover letter. All data is in the paper and interview data cannot be shared as indicated in the cover letter. |  |
| 6. Please review your reference list to ensure that it is complete and correct. If you have cited papers that have been retracted, please include the rationale for doing so in the manuscript text or remove these references and replace them with relevant current references. Any changes to the reference list should be mentioned in the rebuttal letter that accompanies your revised manuscript. If you need to cite a retracted article, indicate the article’s retracted status in the References list and also include a citation and full reference for the retraction notice. | This has been completed. |  |

| ***Reviewers’ Comments*** | | |
| --- | --- | --- |
| *Editor Comment* | *Authors’ response* | *Location in manuscript (page, line number(s))* |
| 1. Is the manuscript technically sound, and do the data support the conclusions?  The manuscript must describe a technically sound piece of scientific research with data that supports the conclusions. Experiments must have been conducted rigorously, with appropriate controls, replication, and sample sizes. The conclusions must be drawn appropriately based on the data presented.  **Reviewer #1: Yes** | Thank you | No change |
| 2. Has the statistical analysis been performed appropriately and rigorously?  **Reviewer #1: N/A** |  | No change |
| 3. Have the authors made all data underlying the findings in their manuscript fully available?  The [PLOS Data policy](https://can01.safelinks.protection.outlook.com/?url=http%3A%2F%2Fwww.plosone.org%2Fstatic%2Fpolicies.action%23sharing&data=05%7C01%7Capace2%40uottawa.mail.onmicrosoft.com%7C00ef3cf4b7554c5848ae08dbe56b5a11%7Cd41fdab17e154cfdb5fa7200e54deb6b%7C1%7C0%7C638356021626348251%7CUnknown%7CTWFpbGZsb3d8eyJWIjoiMC4wLjAwMDAiLCJQIjoiV2luMzIiLCJBTiI6Ik1haWwiLCJXVCI6Mn0%3D%7C3000%7C%7C%7C&sdata=1NMhMZzQ6yLb1Nr%2FuM2uuWzQPdTAdSD08glYTsf%2FGsE%3D&reserved=0) requires authors to make all data underlying the findings described in their manuscript fully available without restriction, with rare exception (please refer to the Data Availability Statement in the manuscript PDF file). The data should be provided as part of the manuscript or its supporting information, or deposited to a public repository. For example, in addition to summary statistics, the data points behind means, medians and variance measures should be available. If there are restrictions on publicly sharing data—e.g. participant privacy or use of data from a third party—those must be specified.  **Reviewer #1: Yes** |  | No change |
| 4. Is the manuscript presented in an intelligible fashion and written in standard English?  PLOS ONE does not copyedit accepted manuscripts, so the language in submitted articles must be clear, correct, and unambiguous. Any typographical or grammatical errors should be corrected at revision, so please note any specific errors here.  **Reviewer #1: Yes** |  | No change |
| 5. Review Comments to the Author  Please use the space provided to explain your answers to the questions above. You may also include additional comments for the author, including concerns about dual publication, research ethics, or publication ethics. (Please upload your review as an attachment if it exceeds 20,000 characters)  Reviewer #1: Thank you for the opportunity to review this scoping review. The authors mapped the literature with regards to the use of virtual Nominal Group Technique (NGT) in healthcare and health education research using a rigorous process. The manuscript is well-written and it was exciting reading through. **I have a few comments for the authors.** Title: **The authors may wish to modify the title slightly as follows: "The Use of Virtual Nominal Groups in Healthcare Research: An Extended Scoping Review". This is because in the methodology, it was stated that only studies that involved healthcare service, health education and guidelines were included. The addition of "healthcare" in the title increases the specificity of the topic.** Abstract: Fair Introduction: The context was well described Objectives: **Just as stated in the title, you may wish to add "healthcare and health education" to the objectives as appropriate and the research questions at the methodology section.** Methodology: Clear. However, **consider the following:** a. Expunge exclusion criteria 1. Since you included only studies that reported on the four key areas of NGT, this exclusion number 1 is not necessary. b. **How many authors participated in the covidence review?** Finally, this is a good paper and in my honest opinion should be considered for publication | We agree and have changed the title to : The Use of Virtual Nominal Groups in Healthcare Research: An Extended Scoping Review".  We have also updated the objectives to reflect the focus on healthcare.  Methodology:  a. we have removed the exclusion criteria as we agree already clear in the inclusion criteria  b. We have added to the text that 3 co-authors and one research assistant participated in the covidence review. | Title  Objectives  Table 1  Last paragraph under Steps 2 & 3 in the methods |
| 6. PLOS authors have the option to publish the peer review history of their article ([what does this mean?](https://can01.safelinks.protection.outlook.com/?url=https%3A%2F%2Fjournals.plos.org%2Fplosone%2Fs%2Feditorial-and-peer-review-process%23loc-peer-review-history&data=05%7C01%7Capace2%40uottawa.mail.onmicrosoft.com%7C00ef3cf4b7554c5848ae08dbe56b5a11%7Cd41fdab17e154cfdb5fa7200e54deb6b%7C1%7C0%7C638356021626348251%7CUnknown%7CTWFpbGZsb3d8eyJWIjoiMC4wLjAwMDAiLCJQIjoiV2luMzIiLCJBTiI6Ik1haWwiLCJXVCI6Mn0%3D%7C3000%7C%7C%7C&sdata=OJKGj%2BlyRWUTW%2BOe8KplU3LUH4WtCu0RC3SRqHALg3k%3D&reserved=0)). If published, this will include your full peer review and any attached files.   If you choose “no”, your identity will remain anonymous but your review may still be made public.   **Do you want your identity to be public for this peer review?** For information about this choice, including consent withdrawal, please see our [Privacy Policy](https://can01.safelinks.protection.outlook.com/?url=https%3A%2F%2Fwww.plos.org%2Fprivacy-policy&data=05%7C01%7Capace2%40uottawa.mail.onmicrosoft.com%7C00ef3cf4b7554c5848ae08dbe56b5a11%7Cd41fdab17e154cfdb5fa7200e54deb6b%7C1%7C0%7C638356021626348251%7CUnknown%7CTWFpbGZsb3d8eyJWIjoiMC4wLjAwMDAiLCJQIjoiV2luMzIiLCJBTiI6Ik1haWwiLCJXVCI6Mn0%3D%7C3000%7C%7C%7C&sdata=mV59ap7Ogwn0nhc3%2F4sEGstbgKq4%2BseHZSTzCZ0L7n8%3D&reserved=0).  **Reviewer #1: No** |  | No Change |
